# ViroFind: A novel target-enrichment deep-sequencing platform reveals a complex JC virus population in the brain of PML patients

**DOI:** 10.1371/journal.pone.0186945

**Published:** 2018-01-23

**Authors:** Spyros Chalkias, Joshua M. Gorham, Erica Mazaika, Michael Parfenov, Xin Dang, Steve DePalma, David McKean, Christine E. Seidman, Jonathan G. Seidman, Igor J. Koralnik

**Affiliations:** 1 Division of NeuroImmunology, Center for Virology and Vaccine Research, Department of Medicine, Beth Israel Deaconess Medical Center, Harvard Medical School, Boston, Massachusetts, United States of America; 2 Department of Genetics, Harvard Medical School, Boston, Massachusetts, United States of America; 3 Department of Neurological Sciences, Rush University Medical Center, Chicago, Illinois, United States of America; The University of Hong Kong, HONG KONG

## Abstract

Deep nucleotide sequencing enables the unbiased, broad-spectrum detection of viruses in clinical samples without requiring an *a priori* hypothesis for the source of infection. However, its use in clinical research applications is limited by low cost-effectiveness given that most of the sequencing information from clinical samples is related to the human genome, which renders the analysis of viral genomes challenging. To overcome this limitation we developed ViroFind, an in-solution target-enrichment platform for virus detection and discovery in clinical samples. ViroFind comprises 165,433 viral probes that cover the genomes of 535 selected DNA and RNA viruses that infect humans or could cause zoonosis. The ViroFind probes are used in a hybridization reaction to enrich viral sequences and therefore enhance the detection of viral genomes via deep sequencing. We used ViroFind to detect and analyze all viral populations in the brain of 5 patients with progressive multifocal leukoencephalopathy (PML) and of 18 control subjects with no known neurological disease. Compared to direct deep sequencing, by using ViroFind we enriched viral sequences present in the clinical samples up to 127-fold. We discovered highly complex polyoma virus JC populations in the PML brain samples with a remarkable degree of genetic divergence among the JC virus variants of each PML brain sample. Specifically for the viral capsid protein VP1 gene, we identified 24 single nucleotide substitutions, 12 of which were associated with amino acid changes. The most frequent (4 of 5 samples, 80%) amino acid change was D66H, which is associated with enhanced tissue tropism, and hence likely a viral fitness advantage, compared to other variants. Lastly, we also detected sparse JC virus sequences in 10 of 18 (55.5%) of control samples and sparse human herpes virus 6B (HHV6B) sequences in the brain of 11 of 18 (61.1%) control subjects. In sum, ViroFind enabled the in-depth analysis of all viral genomes in PML and control brain samples and allowed us to demonstrate a high degree of JC virus genetic divergence *in vivo* that has been previously underappreciated. ViroFind can be used to investigate the structure of the virome with unprecedented depth in health and disease state.

## Introduction

Deep nucleotide sequencing, a process by which a genomic locus is sequenced multiple times, is currently changing the field of clinical virology by providing a platform for the unbiased, broad-spectrum detection of viruses without requiring an *a priori* hypothesis for the source of infection [1, 2, 3]. Nonetheless, the use of deep sequencing for virus detection and discovery in clinical samples is challenged by low cost-effectiveness. Specifically, viral genomes are orders of magnitude smaller compared to the human genome and through deep whole genome sequencing of clinical samples only a small fraction of the sequencing information is related to viral genomes. In addition, viral genomes could be sparse, which also renders the detection of viruses difficult. Detection of viral nucleic acids can be as low as 10 sequences (also referred to as reads) per 25 million total reads generated [4]. To make deep sequencing of viral genomes more cost-effective, we designed a target-enrichment platform, which we named ViroFind. Our in-solution system comprises 165,433 viral probes that cover the genomes of 535 DNA and RNA viruses that are known to infect humans or are considered to be potential zoonotic agents. The ViroFind probes are used in a hybridization reaction to enrich viral sequences out of all the nucleotide sequences present in a clinical sample and therefore enhance the detection of viruses via deep sequencing.

We used ViroFind to investigate the composition of viral populations in brain samples obtained from patients with progressive multi-focal leukoencephalopathy (PML) and from individuals with no known neurological diseases. PML is a demyelinating disease of the brain caused by the polyoma virus JC (JCV), a 5.13Kb double-stranded circular DNA virus [5]. The small and large T antigen are regulatory JCV proteins and comprise the early genes, whereas the viral capsid proteins (VP1, VP2, VP3) as well as agnoprotein are expressed only during the late stage of the viral cycle and comprise the late genes. Expression of the viral genes is driven by a bidirectional noncoding regulatory region (RR), which lies interspersed between the coding parts of the early and late genes. Although JCV is highly prevalent in the human population, only a small subset of immunocompromised individuals develops PML [6]. It is likely that rearrangements of the regulatory region confer enhanced virulence, by up-regulating transcription of viral genes, and MAD-1 is the prototypical PML-associated JCV strain with rearranged regulatory region [5, 7, 8]. Previously, dideoxynucleotide sequencing (also referred to as Sanger sequencing) has been used to study the evolution of the regulatory region from the archetype virus to PML-associated variants [9, 10]. However, Sanger sequencing does not detect minority viral variants that might contribute key genetic rearrangements and it provides a biased view of the dynamics of viral populations [11, 12]. More recently, the regulatory region of JCV from plasma and cerebrospinal fluid (CSF) samples of PML patients was analyzed via deep sequencing and a highly dynamic process of RR reorganization was observed. In addition, archetype JCV, thought to be non-pathogenic and usually found in the kidney and urine, was rarely detected in the CSF compartment [13]. Hence, although reorganization of the noncoding region might play a significant role in viral gene expression and virulence, other genomic loci are likely to contribute to viral pathogenesis.

The viral capsid protein VP1 binds to sialic acid cell receptors and is involved in viral cell entry [5]. Based on *in silico* and *in vitro* work, specific VP1 nucleotide substitutions are thought to alter the ability of JCV to bind to sialic acid and hence these mutations likely affect tissue tropism [14, 15]. Here, we use our target-enrichment platform to detect and analyze all the JCV populations in brain tissue from PML patients with unprecedented depth. We show that in the brain of PML patients, multiple JCV variants constitute highly complex viral populations. We identify *in vivo* VP1 mutations that could impact viral tropism and be linked to the development of PML. Lastly, we demonstrate that JCV genomic fragments can be detected in the brain of individuals with no known neurological disease, which is consistent with JCV latency in the human brain. Collectively, our findings add to our understanding of JCV biology and PML pathogenesis and our work establishes a target-enrichment deep-sequencing platform for clinical applications such as enhanced virus detection and analysis of viral genetic structure and variation.

## Results

### Enhanced detection of JC virus sequences with ViroFind

**Fig 1:**
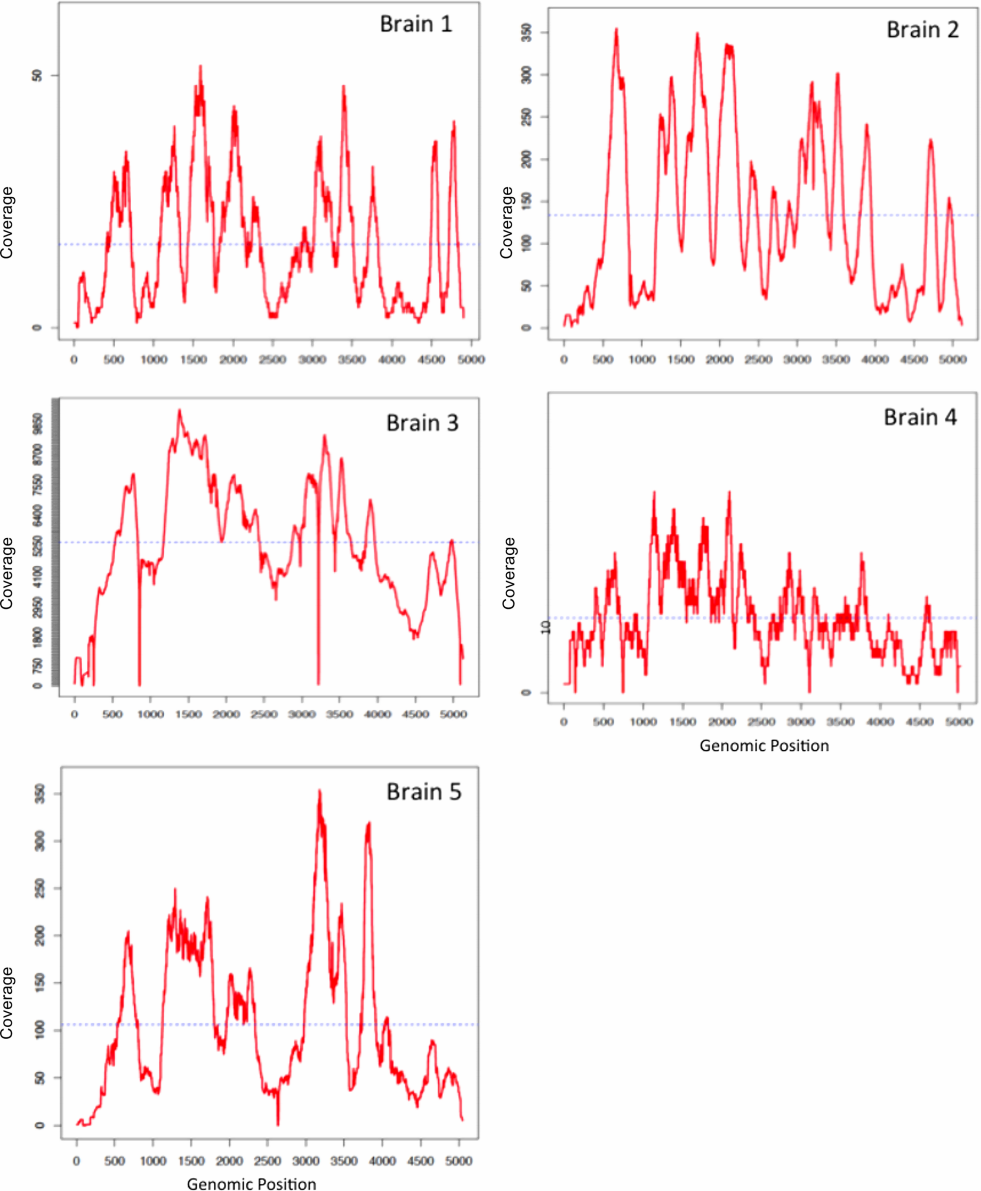
Coverage plots of JC virus genome in brain samples from PML patients. Coverage plots of JC virus genome in brain samples from 5 PML patients. The figure shows the number of times each nucleotide of the JC virus genome was read during the sequencing process (coverage). By using ViroFind we achieved high coverage of the JC virus genome, which is required for the reliable identification of all JC virus variants.

We first extracted DNA from the five PML brain samples and used the nucleic acid to construct libraries, which were directly sequenced using the Illumina MiSeq platform. We were only able to detect a small number of unique JC virus sequences per sample, ranging from 7 JCV reads per 520,848 total reads to 2,940 JCV reads per 428,468 total reads (Table 1). We then used the same DNA libraries for a ViroFind hybridization reaction. The output of the reaction was sequenced via MiSeq. We observed a substantial increase in the number of unique JCV reads detected per PML brain sample, ranging from 584 viral reads per 1,000,280 total reads to 375,653 viral reads per 430,842 total reads. The enrichment of JC virus sequences attributable to ViroFind ranged from 33 to 127-fold. Interestingly, we observed that the number of viral sequences captured by ViroFind increased concordantly with the number of viral sequences detected by direct deep sequencing, as shown in Table 1. We performed computational analyses to calculate the number of times each viral nucleotide position was sequenced, which is termed coverage. Adequate sequencing coverage, typically at least 10X, is key for reliably identifying viral variants given that sequencing errors can result in false variant calling [16, 17]. For all PML brain samples, with the exception of brain sample 4 and small genomic regions in brain sample 1, we achieved at least 10X coverage for all genomic positions (Fig 1). We also used a robust and established variant-calling algorithm which accounts for sequencing errors. (V-phaser 2, Broad Institute) [18]. This approach allowed us to reliably identify all the JCV variants present in the PML brain samples.

**Table 1:** ViroFind performance characteristics.

Sample	Platform	Mapped JCV Reads	Total Reads	Mapped Reads/Total Reads	# Fold Enrichment
PML Brain sample 1	ViroFind	1,097	1,023,903	1 x 10^−3^	33
	Deep Sequencing	24	738,541	3 x 10^−5^	
PML Brain sample 2	ViroFind	9,327	1,826,482	5 x 10^−3^	58
	Deep Sequencing	45	515,119	8 x 10^−5^	
PML Brain sample 3	ViroFind	375,653	430,842	9 x 10^−1^	127
	Deep Sequencing	2,940	428,463	7 x 10^−3^	
PML Brain sample 4	ViroFind	584	1,001,280	6 x 10^−4^	43
	Deep Sequencing	7	520,848	1 x 10^−5^	
PML Brain sample 5	ViroFind	10,345	1,345,674	7 x 10^−3^	116
	Deep Sequencing	56	899,654	6 x 10^−5^	

The table shows the enrichment of JC virus sequences attributable to ViroFind in five brain samples from PML patients. We extracted DNA from brain tissue and made sequencing libraries that were used for a deep sequencing experiment to detect JC virus sequences. We then used the same libraries for a virus capture reaction (ViroFind). The number of unique viral reads per total number of reads increased substantially when using ViroFind and the enrichment of JC virus reads attributable to our platform was between 33–127 fold.

### Detection of multiple JC virus variants in PML brain samples

We first looked for nucleotide substitutions in the JCV VP1 region, which spans nucleotide positions 1,469 to 2,533 of the viral genome. Capsid protein VP1 interacts with sialic acid receptors on cell surface and facilitates cell entry of the virus [5]. Hence, VP1 mutations leading to amino acid substitutions could be associated with conformational changes of the protein and alter the binding affinity to sialic acid receptors. We found multiple VP1 nucleotide substitutions, compared to the consensus sequence, in all PML brains except brain 4, which was sequenced in lower coverage than the other samples (Table 2). The total number of JCV VP1 variants observed in all brain samples was 24 and 12 of these variants encoded amino acid changes, whereas the other 12 VP1 nucleotide substitutions were silent. By examining all mutations associated with amino-acid substitutions we found that multiple changes in residues located within the sialic acid binding site such as VP1 codons 55, 60, 66, 128, 134. Interestingly, mutation D66H was observed in 4 of 5 (80%) brain samples and it represented the dominant viral variant in two brain samples. Amino-acid substitution D66H, where an acidic residue (aspartic acid) is substituted by a basic one (histidine) has been shown to maintain the ability of the virus to bind to sialic acid receptors, unlike amino acid changes in other codons, including 55 and 60, where the binding capacity is dramatically reduced [14, 15]. In addition, in the VP2 genomic region we detected one missense mutation, R322S, in PML brain 3, which has been previously described, and several nonsense coding point mutations [18]. Lastly, we only detected nonsense coding point mutations in the VP3 genomic region.

**Table 2:** JC virus VP1 capsid protein variants isolated from PML brains.

Sample	Nucleotide position	Consensus	Variant	Variant percentage	Amino acid substitution
Brain 1	1632	A	T	21	L55H
	1654	T	A	16	
	1664	C	G	19	D66H
	2224	G	A	16	
	2227	T	C	16	
	2266	A	G	14	
Brain 2	1648	G	T	6	K60N
	1648	G	C	9	K60N
	1664	G	C	47	H66D
	1753	A	T	39	
	1786	G	A	42	
	1804	T	C	46	
	1850	G	A	42	T128A
	1869	G	C	45	A134G
	1959	A	C	32	T164K
	2293	G	C	29	
	2428	A	G	45	
	2429	A	G	44	V321I
	2446	T	G	42	
	2462	C	G	41	E332Q
	2524	A	G	49	
	2539	A	G	49	
Brain 3	1664	G	C	42	H66D
	1986–1987	A, T	2-base deletion	1	
Brain 5	1664	C	G	21	D66H

The table shows all the nucleotide substitutions in the JC virus VP1 gene along with corresponding amino-acid changes and the percentage of each JCV variant within the total viral population. ViroFind enabled us to achieve high coverage of the entire JC virus genome in the brain of PML patients and, for the first time, uncover the complex genetic structure of the viral population in each sample. We demonstrate that more than one JCV variants are frequently found in the brain of PML patients and that a subset of JCV VP1 variants encode for specific amino acid substitutions, which could impact viral capsid protein conformation and consequently tissue tropism. (No VP1 variants were found in brain sample 4).

**Fig 2:**
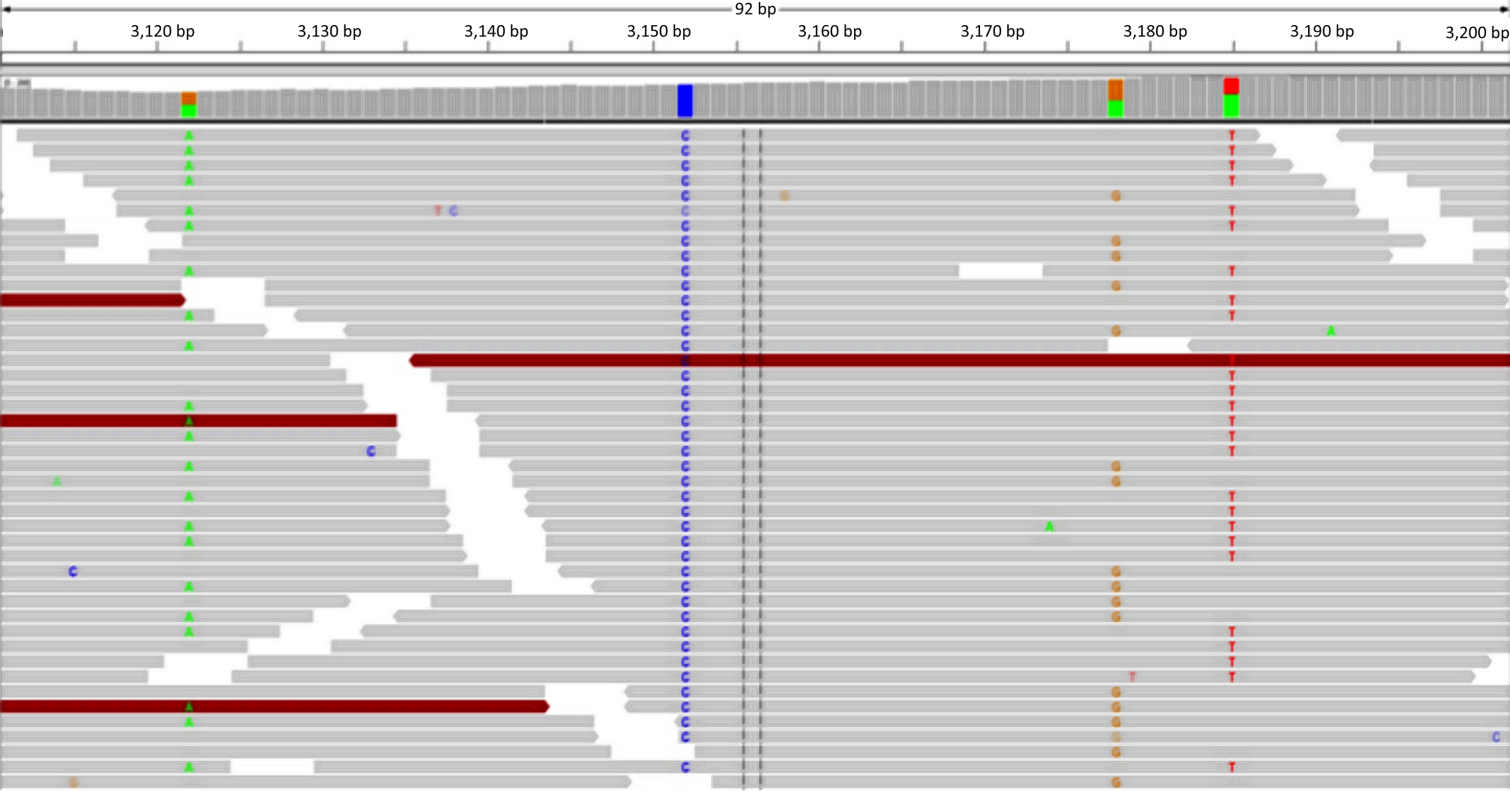
Multiple JC virus variants within the same PML brain sample. Visual inspection of the JC virus 75 base-pair nucleotide reads reveals multiple single nucleotide polymorphisms (SNP). Each read is represented by a single horizontal arrow. SNPs in positions 3,122, 3,178 and 3,185 of the JC virus genome occur independently, which is consistent with the presence of at least three distinct viral variants in the same PML brain.

We then visually inspected different genomic areas of the viral genome by using the Integrative Genomics Viewer (Broad Institute). Visual inspection of the 75 base-pair nucleotide reads revealed multiple single nucleotide polymorphisms (SNP) within each sample. Fig 2 shows an example of multiple SNP in three neighboring nucleotide positions (3,122, 3,178 and 3,185) of the viral genome in the second PML brain. Each pair of the three SNP appears within different reads, which is consistent with independent nucleotide substitutions and hence, with the presence of three distinct viral variants in the same PML brain. This is in line with our results of multiple JC virus variants in each sample. Lastly, we found that the regulatory region sequences in all five PML brains aligned against the prototypical tandem repeat pattern consistent with CNS isolates of JCV.

### Genetic distance between the JC virus sequences of each PML brain sample and MAD-1

**Fig 3:**
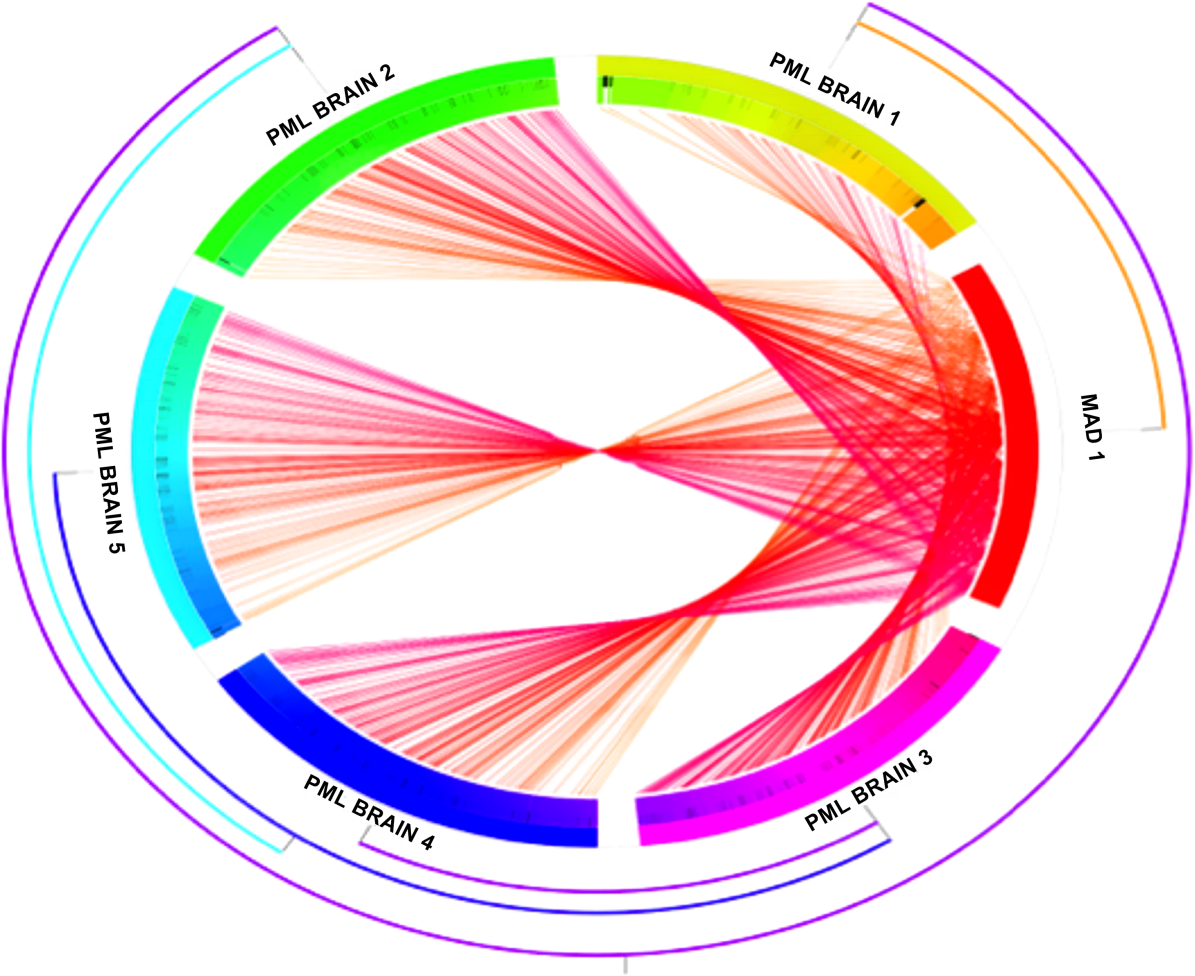
Genetic distance between the JC virus sequences of each brain sample and MAD-1 sequence. The figure illustrates the genetic divergence between the JC virus sequences found in each PML brain and MAD-1 (red bar), the JC virus prototype sequence associated with PML. Each chord corresponds to a single nucleotide polymorphism. The presence of multiple JCV variants within each PML brain accounts for a remarkable degree of genetic variation and suggests that the genetic complexity of JC virus in the brain compartment has previously been underestimated.

We then analyzed the JCV variants in genomic locations other than VP1 (VP2, VP3, agnoprotein, large and small T antigen) and we calculated the genetic distance between the JCV isolates and Mad-1, as shown in Fig 3. We found a remarkable degree of intra-patient genetic divergence due to multiple single nucleotide substitutions in all genomic regions. We also identified 70 variants with a frequency above 1% within the viral population, among all 5 brain samples, 57 of which were silent and 13 nucleotide substitutions led to amino acid changes. In addition, the consensus sequences of each brain sample were genetically distinct from the prototypical PML-associated JCV variant, MAD-1. This degree of JCV genetic variation of coding regions in the brain has not been previously reported.

### Detection of JC virus and human herpes virus 6 sequences in the brain of individuals with no known neurological disease

Lastly, we used ViroFind to investigate the presence of any viral sequences in 18 control brain samples from individuals with no known neurological disease. We were able to detect sparse JC virus fragments in 10 of 18 (55.5%) of control samples and sparse human herpes virus 6 B (HHV6B) sequences in the brain of 11 of 18 (61.1%) control subjects. Specifically up to 1761 bp (37.64%) of the JC virus genome was covered in control brains (176 viral reads per 2,150,667 total reads). In addition, we were able to assemble up to 44,419bp (27.40%) of the HHV6 genome (3,450 reads per 4,005,456 total reads). The prevalence of JCV sequences in non-PML brains is within the range of those reported in other studies based on molecular PCR, targeting a specific JCV genomic region [19–23]. The prevalence of HHV6 sequences in control brains exceeded the previously reported prevalence of 50% [24, 25]. These results were confirmed by JCV and HHV6 specific quantitative PCR.

## Discussion

Deep sequencing is revolutionizing the field of clinical virology by overcoming many of the limitations of other molecular diagnostic tools such as targeted molecular PCR and Sanger sequencing [2]. Molecular PCR is pathogen-specific and genomic loci-specific whereas Sanger sequencing offers a limited view of the virome given that viral variants below 20–30% of the total population are not detected [11, 12]. Hence, deep sequencing enables the unbiased, broad-spectrum detection of viral genomes but increased sequencing depths are needed for the analysis of genetic structure and variation [26]. This is largely because the genome of most of the human viruses ranges from 1 to 240 x 10^3^ base-pairs (bp) and it is much smaller compared to the human genome (3 x 10^9^ bp). Hence, most of the sequencing information of clinical samples is related to the human genome. Nonetheless, to assemble complete viral genomes and identify viral variants a high number of unique viral reads is needed. To address these limitations, we designed ViroFind, a target-enrichment platform for the unbiased detection of viruses. ViroFind selects for the viral nucleic acids prior to the deep sequencing experiment and therefore enriches the viral sequences of interest. We paired ViroFind with our published bioinformatics pipeline for the detection of pathogenic nucleic acid sequences in next generation sequencing data and we developed a complete system for the analysis of viral genomes in clinical samples [27]. As opposed to other in-solution sequencing platforms for the capture of viral nucleic acid, we focused on DNA and RNA viruses that are known to infect humans or could cause zootonic diseases, aiming at producing a diagnostic tool with direct clinical applications and the potential for commercial use [4, 28]. The size of our viral probe system does not exceed 10 Mega base pairs (Mbp), which reduces the production cost substantially. We used ViroFind to enrich JC virus sequences from human brain samples and investigate the genomic structure of the entire viral genome with unprecedented depth.

First, we discovered that multiple JCV variants constitute the intra-host viral population in PML brains and that mutations are in fact interspersed throughout the viral genome. This degree of genetic variation indicates a striking, dynamic evolution of the viral genome in the brain compartment that has been previously underestimated. By using ViroFind we were able to achieve high sequencing depth, reliably identify viral variants and analyze the entire viral genome, unlike previous sequencing studies that were primarily limited to the regulatory region of the virus or were based on Sanger sequencing [8–10, 13]. Although this degree of intra-host genomic variation is rather surprising for a DNA virus, deep sequencing reports of other specific DNA viruses, such as the human cytomegalovirus, have uncovered high degree of intra-host genetic divergence, at times comparable to RNA viruses [29, 30]. Our findings suggest that viral replication in PML brains occurs in a rate that allows for multiple mutations but we cannot exclude that some of these mutations originate from other compartments. Previous reports suggest a low mutation rate of JC virus in the renal compartment, where the virus resides in asymptomatic individuals, however multiple JCV variants have been detected in the blood and cerebrospinal fluid of PML patients [13, 31–33]. It is overall possible that genomic evolution occurs in peripheral sites of viral replication and becomes even more dynamic in the central nervous system of individuals suffering from PML.

Second, we focused on the genomic analysis of the viral capsid protein VP1 gene. Although the analysis of other genomic loci, primarily of the non-coding regulatory region, has been previously emphasized, it has also been proposed that VP1 genomic evolution gives rise to JCV variants with enhanced virulence [14, 15]. This is based on amino acid substitutions sufficient to confer conformational changes in the site of VP1 interactions with sialic acid receptors. Both *in silico* and *in vitro* data support that amino acid substitutions in VP1 codons 55, 60, 61, 66, 267, 128, 134, 269, 271 alter the binding affinity of VP1 to its receptors, which likely impacts tissue tropism, and in fact these mutations have been detected in CSF and plasma, but not urine, obtained from PML patients [14, 15, 33]. We were able to detect 24 VP1 single nucleotide substitutions in 5 PML brain samples, 12 of which led to amino acid changes, including codons 55, 60 and 66. Interestingly, mutation D66H was present in 4 of 5 (80%) of PML brain samples and it represented the dominant variant in two of these brain samples. The substitution of aspartic acid (D) by histidine (H) in VP1 amino acid location 66 is likely associated with a structural modification of the capsid protein but it is the only amino acid substitution that does not affect *in vitro* binding affinity of the JCV variant to sialic acid receptors, including receptors located on glial cells [14, 15]. On the contrary, all other studied mutations seem to decrease the binding capacity of the virus to different cell types. It is therefore possible that D66H is a key substitution in the etiopathogenesis of PML by providing a viral fitness advantage to the JCV variant. Based on our findings and previously published data, we propose that specific VP1 mutations, such as D66H, offer an evolutionary advantage and could act in concert with other virus-related or host-related factors to account for the development of PML. In this model, key mutations might arise in the periphery or evolve within the central nervous system once viral proliferation occurs in this compartment. Furthermore, VP1 mutations could be associated with viral escape from an antibody-mediated immune response against the virus, given that anti-JCV antibodies might not interact effectively with newly formed VP1 mutants [34–36]. In sum, further understanding of the intra-host adaptive changes of JC virus VP1 will shed light to viral pathogenesis and possibly provide a foundation for the development of therapeutic agents against JC virus, such as broadly neutralizing antibodies that interact will all VP1 variants.

Third, we investigated the presence of viral sequences in 18 control brain samples from individuals with no known neurological disease. We detected sparse JC virus sequences in 10 of 18 (55.5%) brain samples, which is consistent with the viral prevalence reported in other PCR-based studies, which ranges from 26% to 71% [19–23]. We also detected sparse human herpes virus 6B (HHV6B) sequences in the brain of 11 of 18 (61.1%) control subjects. HHV6B has been detected in the brain and in other organs of asymptomatic individuals and it has been proposed that expression of HHV6 proteins might be linked to neurological diseases such as multiple sclerosis [37]. We therefore demonstrated that ViroFind enhances the ability to detect viruses, compared to conventional methods and to deep-sequencing samples directly. The detection of very sparse JC virus genomic fragments in control brains is consistent with the fact the JC virus establishes latency in the central nervous system and it is highly unlikely that it signifies active viral replication [2]. Whether, under specific circumstances such as immune dysfunction, productive infection originates from the brain-resident virus or from viral trafficking from the periphery is currently not known, although these two possibilities are not mutually exclusive.

In conclusion, our work establishes ViroFind as a target-enrichment deep-sequencing platform for the enhanced broad-spectrum detection of viral sequences in clinical samples with the potential of commercial use. We demonstrate how ViroFind can be used to address long-standing questions in the field of polyoma viruses and progressive multifocal leukoencephalopathy, a devastating demyelinating disease of the brain. Specifically, our viral genome-wide findings uncover a highly complex JC virus population in the brain of patients with PML and pinpoint to specific mutations in the viral capsid protein VP1 gene which are likely to impact viral tropism and virulence. Our findings suggest that further understanding of the viral intra-host evolution of VP1 and potentially of other genomic loci are key for fully elucidating JC virus pathogenesis in the human host. Uncovering this degree of genetic polymorphism of JC virus *in vivo* would not have been possible without the ViroFind-based high-resolution view into viral genetic structure and variation. Lastly, the detection of viral sequences in brain samples from asymptomatic individuals suggests that deep-sequencing target-enrichment platforms are a sensitive tool for studying the composition of the virome in healthy individuals. Given that a multitude of viral diseases originate from resident intra-host viruses, knowledge of the human virome structure is the first step in understanding disease pathogenesis.

## Materials and methods

### Ethics statement

The Institutional Review Board (IRB) at the Beth Israel Deaconess Medical Center (IRB) approved this study. We obtained an inter-institutional material transfer agreement and obtained brain samples from the University of Texas and the University of Hawaii. All samples were anonymized.

### Clinical samples

We collected 5 post mortem PML brain samples from HIV-infected individuals and 18 control brain samples from individuals without HIV or PML, both from BIDMC and the other two institutions. PML patients were diagnosed based on clinical and neuroradiological criteria and confirmed by histological examination. Brain fragments containing PML lesions were selected for analysis. Control brain samples were obtained from individuals with no known neurological disease.

### ViroFind design

We downloaded all the viral genomes from the National Center for Biotechnology information (NCBI) in March 2015 (http://www.ncbi.nlm.nih.gov). We selected 535 DNA and RNA viruses that are known to infect human or potentially cause zoonosis (see S1 Table). These genomes represent reference sequences. We aligned the viral genomes of interest against the human genome and we removed the overlapping sequences of human endogenous retroviruses and of short interspersed elements (SINEs) via a process referred to as masking (RepeatMasker, Institute for Systems Biology). By masking these sequences, we aimed at excluding them from the set of viral probes and hence from selecting them from clinical samples during the wet lab experiments. We then used a proprietary computational approach (SureDesign, Agilent Solutions, Santa Clara, CA) to design the ViroFind probes, derived from the viral sequences. Each viral probe is a 125 bp biotinylated RNA molecule, complementary to a viral genomic region. It was at the discretion of the manufacturer, based on proprietary algorithms, to redistribute probes for target base coverage and redundancy. Each base was covered at least once (1X tiling). The design of the probes is based on the GC content of each particular genomic area (high GC content might allow for non-specific binding between nucleic acids) and the ability of the specific probe to hybridize with complementary sequences under the conditions of the hybridization reaction. The GC content of the ViroFind probes ranges from 43 to 68%. The manufacturer provided us with the genomic coordinates of the probes and we first performed *in silico* experiments to confirm adequate capture of viral sequences. The *in silico* target base coverage was 91% and 95% using 0-bp-offset and 100-bp-offset metrics respectively. The total number of ViroFind viral probes is 165,433 (9.756 Mbp) and each probe is represented in the millions in each hybridization reaction. Coverage of the viral genomes by the probe set ranges from 83% to 100% of the viral genome and for 90.61% of the viruses, 100% of the genome is covered (S1 Table). A tab-delimited text file (BED file) with the ViroFind probe genomic coordinates is available for download at ViroFind BED file.

### Construction of sequencing libraries

We extracted DNA and RNA from the fronzen brain samples by using a spin-column method as previously described (Qiagen DNeasy/RNeasy Blood and Tissue Kit, Germantown, MD) [38]. Total RNA was reverse transcribed to complementary DNA (cDNA) by using the Superscript III First-Strand Synthesis System (Invitrogen, Carlsbad, CA) [39]. Both DNA and cDNA were used as input material for the DNA and cDNA library construction (1000 ng input material respectively) using the Agilent library kit (SureSelect^XT2^ Target Enrichment system for Illumina paired-end multiplexed sequencing, Agilent Solutions, Santa Clara, CA). First, we fragmented the nucleic acid using a Covaris sonicator to 250 bp fragments. We then used the end-repair enzyme at 20°C for 30 minutes to repair the ends of the fragmented DNA/cDNA molecules. Next, we performed a cleanup step using AMPure XP beads to purify the end-repaired nucleic acids and we then proceeded with adenylation of the 3’ end of the DNA/cDNA molecules using dA-Tailing reagent of the Agilent library kit. We then ligated adaptors to the nucleic acid molecules of each library. The adaptors serve a dual purpose: **1)** they include unique 8bp sequences (indexes) which help bar-code and multiplex different samples in one sequencing experiment, and **2)** they include primer sequences for the PCR amplification steps needed downstream. Lastly, we used a herculase DNA polymerase and primers complementary to the indexes (included in the Agilent kit) to perform 5 cycles of PCR. We then repeated a purification step using the AMPure XP beads and quantified the library material using the Agilent 2200 tape-station (Agilent Solutions, Santa Clara, CA).

### Hybridization reaction

For the ViroFind hybridization reaction we pooled the brain library samples to 1500ng total mass in 7 μL volume using a vacuum concentrator. We then added 9 μL of the SureSelect blocking mix which includes blockers designed to prevent non-specific annealing between the adaptor molecules and kept the solution at 65°C for 5 minutes. Next, we added 5 μL of the ViroFind probes, the SureSelect hybridization buffer reagent and the RNAase block reagent. The latter prevents degradation of the RNA ViroFind probes during the hybridization reaction. We then allowed an overnight (18h) hybridization reaction to occur at 65°C. At this point, any complementary to the probes sequences from the sample libraries hybridize in the solution. The next day, we washed six times the hybridization products with Dynabeads MyOne Streptavidin T1 magnetic beads which were brought at a temperature of 65°C. During the washing steps, we aimed at discarding nucleic acids unbound to the ViroFind probes. Specifically, the ViroFind probes are biotinylated and biotin has an affinity for streptavidin, which is magnetized by the beads. As a net result, by using a magnet during the washing steps, we magnetically pulled down the hybridized molecules from all the library molecules. Next, we performed a quantitative PCR to visualize the amplification curve and use the number of cycles of only the exponential phase of the PCR curve for a post-capture amplification step. We then proceeded with PCR amplification of the post-hybridization product and with another AMPure beads cleanup step. Lastly, we quantified our product using again the Agilent tape-station and we submitted our sample for deep sequencing.

### Deep sequencing and bioinformatics pipeline

We used the Illumina MiSeq instruments available at the Harvard Biopolymers facility to perform paired-end deep sequencing. The output of one Miseq V3 experiment is 20–30 million total reads. Once we received the raw sequencing data we performed a quality check and discarded reads that do not pass quality filters [40]. We then use the unique sequences of the indexes to de-multiplex the clinical samples. Lastly, we trimmed the adaptor sequences from all reads.

To detect and analyze viral sequences in the sequencing data we relied on our previously published computational pipeline [27]. Given the need to handle big sequencing data sets and computationally intense algorithms, we performed our analysis on *Orchestra*, the Harvard Medical School high performance computational environment. Briefly, we first align our reads against the human genome and we discarded any reads that aligned to the human genome. We then aligned the remaining reads against the NCBI dataset of all viral genomes. The output file is a tab-delimited text file that contains sequence alignment data, referred to as SAM file and its binary version, referred to as BAM file. These files include information on all mapped reads and can be used to visualize viral reads. We also used the V-phaser 2 software module (V-phaser 2, Broad Institute) to identify viral variants (variant calling) as previously described [26]. Importantly, the differentiation of low-frequency variants from sequencing errors can be challenging and high coverage of a specific genomic location is needed in order to reliably call variants. By using ViroFind, we achieved substantial enrichment of viral sequences and hence adequate sequencing depth for the reliable identification of true variants. We used Picard tools (Picard tools, Broad Institute) to mark and remove PCR-derived duplicate reads from the sequencing data and hence work only with unique viral reads (http://picard.sourceforge.net). Lastly, We used the DNAStar software package (DNAStar, Lasergene) to calculate the genetic distance between viral variants and reference sequences (www.dnastar.com). Specifically, we first used the MegAlign tool to align the JCV sequences from the PML samples against the MAD-1 sequence by the Clustal W method. We then exported the alignments as a GenVision project with the following software parameters: chord alignment, minimum length 15, minimum sequence identity 50%, root outside. The output graph illustrates the genetic distance of all single nucleotide polymorphisms from the prototypical MAD-1 sequence.

## Supporting information

S1 Table List of DNA and RNA viruses included in ViroFind along with coverage of the viral genomes. (XLSM)
